# Novel Lipidated Imidazoquinoline TLR7/8 Adjuvants Elicit Influenza-Specific Th1 Immune Responses and Protect Against Heterologous H3N2 Influenza Challenge in Mice

**DOI:** 10.3389/fimmu.2020.00406

**Published:** 2020-03-10

**Authors:** Shannon M. Miller, Van Cybulski, Margaret Whitacre, Laura S. Bess, Mark T. Livesay, Lois Walsh, David Burkhart, Hélène G. Bazin, Jay T. Evans

**Affiliations:** ^1^Center for Translational Medicine, University of Montana, Missoula, MT, United States; ^2^Division of Biological Sciences, University of Montana, Missoula, MT, United States; ^3^Department of Biomedical and Pharmaceutical Sciences, University of Montana, Missoula, MT, United States

**Keywords:** adjuvant, Vaccine, influenza, TLR4 (Toll-like receptor 4), Influenza challenge model, precision vaccines, TLR7/8 agonists

## Abstract

Most licensed seasonal influenza vaccines are non-adjuvanted and rely primarily on vaccine-induced antibody titers for protection. As such, seasonal antigenic drift and suboptimal vaccine strain selection often results in reduced vaccine efficacy. Further, seasonal H3N2 influenza vaccines demonstrate poor efficacy compared to H1N1 and influenza type B vaccines. New vaccines, adjuvants, or delivery technologies that can induce broader or cross-seasonal protection against drifted influenza virus strains, likely through induction of protective T cell responses, are urgently needed. Here, we report novel lipidated TLR7/8 ligands that act as strong adjuvants to promote influenza-virus specific Th1-and Th17-polarized T cell responses and humoral responses in mice with no observable toxicity. Further, the adjuvanted influenza vaccine provided protection against a heterologous H3N2 influenza challenge in mice. These responses were further enhanced when combined with a synthetic TLR4 ligand adjuvant. Despite differences between human and mouse TLR7/8, these novel lipidated imidazoquinolines induced the production of cytokines required to polarize a Th1 and Th17 immune response in human PBMCs providing additional support for further development of these compounds as novel adjuvants for the induction of broad supra-seasonal protection from influenza virus.

## Introduction

The connection between the innate andadaptive immune system is instrumental for eliciting protective, durable, vaccine-elicited protection against infectious diseases. Current seasonal influenza virus vaccines can be effective if well-matched to the circulating strains; however, mismatch between vaccine strains and circulating strains, particularly in the case of H3N2 (1), leads to a sharp drop in vaccine effectiveness (2, 3). Further, recent analyses have indicated that intraseasonal waning immunity against seasonal influenza is significant, and reductions in vaccine effectiveness may occur more rapidly for H3N2 than for H1N1 strains (4–8). Along with neutralizing antibodies [reviewed in (9)], previous studies have demonstrated that protection against influenza correlates with pre-existing levels of influenza-specific Th1-type CD4 T cells (10) and that passively transferred Th1 or Th17 memory T cells can protect naïve mice against influenza (11). However, current licensed seasonal and pandemic influenza vaccines fail to elicit efficient T cell responses (12).

One solution to this challenge is to develop novel adjuvants that target distinct innate immune receptors and trigger the required innate immune response to subsequently shape the desired adaptive immune response. Thus far, defining the required innate immune stimulators, or adjuvants, to subsequently elicit a protective, durable T cell response has been difficult. Promisingly, new vaccine adjuvants based on Toll-like receptor (TLR) ligands have been approved for human use including monophosphoryl lipid A, a TLR4 agonist and a component of the clinically approved adjuvant systems AS01 and AS04 (13–17), and CpG, a TLR9 agonist (18, 19). In the case of pandemic influenza virus vaccines, emulsion based adjuvant systems such as AS03 [reviewed in (15)] and MF59 [reviewed in (20)] have proven safe and effective at inducing strong humoral immunity to the matched vaccine and challenge strains. However, we still lack an influenza vaccine capable of eliciting consistent protection against drifted influenza strains. Two particularly promising adjuvant targets are TLR4 and TLR7/8. TLRs recognize various bacterial and viral components [reviewed in (21)]. TLR7 and TLR8 are expressed in the endosome and specifically recognize single stranded RNA (ssRNA) (22, 23). As the influenza virus is a single-stranded RNA virus and is recognized by TLR7/8 amongst other pattern recognition receptors (PRRs) (24), TLR7 and TLR8 are attractive targets for influenza virus vaccine adjuvants. Ligation of TLR7/8 elicits production of pro-inflammatory cytokines, such as TNFα, as well as the anti-viral cytokine IFNα, and induces upregulation of co-stimulatory molecules on antigen-presenting cells (APCs) that are critical for enhancing antigen-specific T cell responses (25–27). The anti-viral effects of TLR7/8 agonists are primarily generated through TLR7 ligation in plasmacytoid dendritic cells (pDCs) and their secretion of IFNα (25, 26) while pro-inflammatory responses generated through TLR8 ligation in myeloid dendritic cells [mDCs; (28, 29)] help shape the resulting innate and adaptive immunity. TLR4 has several known ligands, the most well-known of which is lipopolysaccharide (LPS). TLR4 is unique in that it can signal via the MyD88 pathway when TLR4 is engaged on the cell surface, resulting in production of proinflammatory cytokines [reviewed in (30)], whereas endosomal TLR4 signals via the TRIF pathway and induces the production of type I interferons (31, 32), which are critical for anti-viral immune responses, and pro-IL-1β (33–35).

TLR7/8-based adjuvants have a long history of efficacy in a pre-clinical and clinical setting. Early investigations into first generation TLR7/8 agonists as vaccine adjuvants, such as R848, demonstrated high reactogenicity when administered orally or intravenously limiting their widespread use (36). Novel TLR7/8 agonists which do not result in systemic immune responses but nevertheless activate the innate and, subsequently, the adaptive immune system, are of great interest. Recently, several groups have developed lipidated or alum adsorbed TLR7/8 ligands to overcome the rapid systemic distribution and toxicity noted with the previous compounds (37–44). Additionally, previous work has demonstrated that the combination of a TLR4 agonist with a TLR7/8 agonist leads to synergistic upregulation of IFNγ, IL-12p70, and IFNα (45–49), cytokines which induce and enhance Th1 type immune responses that are particularly effective at controlling viral infections [reviewed in (50)].

We have previously reported on the discovery and activity of core (non-lipidated) TLR7/8 agonists, one of which enhanced humoral and cell-mediated immunity to the CRM197 mutant diphtheria toxin protein in pigs (51). Here, we build upon these studies by reporting on novel lipidated imidazoquinoline TLR7/8 ligands, alone or in combination with a synthetic TLR4 ligand, and their ability to elicit strong antigen-specific humoral and Th1- or Th17-mediated T cell responses to a co-administered seasonal split H3N2 influenza vaccine in mice. We found that lipidated imidazoquinolines TLR7/8 agonists can elicit a Th1-biased influenza specific immune response in mice and when combined with a TLR4 agonist, elicit a Th17 response as well. Further, the adjuvanted vaccine-induced adaptive immune responses provided durable protection from a heterosubtypic H3N2 influenza virus challenge, particularly in the case of a combination TLR4 and TLR7/8 adjuvant. When tested in human PBMCs, these adjuvants were able to elicit a cytokine profile suggestive of Th1- and Th17-polarization.

## Reagents

### TLR7/8 Agonist Compounds and Formulation

CRX-601 (TLR4 agonist) (52), UM-3001 (non-lipidated TLR7/8 agonist) (53), and UM-3003, -3004, and -3005 (54) were synthesized following established procedures and formulated in 2% glycerol in water as previously reported (51). Physical characteristics are presented in Table S1.

### HEK293 TLR7 and TLR8 Reporter Assays

Human TLR7 or TLR8 and mouse TLR7 or TLR8 expressing HEK cells were obtained from Invivogen (San Diego, CA) or Novus (human TLR7 only). Cells were cultured according to the manufacturer's instructions in DMEM with 10% FBS and selection antibiotics. HEK cells were plated at a density of 3 × 10^5^ cells/well in a flat bottom 96 well-plate and incubated for 18–24 h at 37°C with indicated concentrations of various TLR7/8 agonists. Cell supernatants were harvested and analyzed for NFκB via the manufacturer's instructions using the QuantiBlue kit (Invivogen). SEAP activity was assessed by reading the optical density (OD) at 620–655 nm with a microplate reader. Data are expressed as the fold change in OD over vehicle treated cells.

### Human Peripheral Blood Mononuclear Cell (PBMC) Isolation and Stimulation

Human blood was obtained from healthy adult donors through a University of Montana Institutional Review Board (IRB)-approved protocol. PBMCs were separated from whole blood via density gradient separation using Histopaque 1,077 (Sigma). For PBMC-based assays, cells were resuspended at the desired cell concentration in complete media (RPMI1640+10% FBS+antibiotics). Cells were treated with the indicated compound concentrations and stimulated for 6–24 h depending on the assay as indicated in the figure legends and assays outlined below.

### PBMC Cytokine Analysis

For Luminex assays, supernatants were harvested from treated human PBMCs following 18–24 h of incubation. Supernatants were analyzed using Luminex multiplex panel for analytes IL-1β, IL-12p70, IL-23, IL-6, TNFα, IFNα, and IL-4 (R&D Systems) per the manufacturer's instructions. Multiplex analysis was performed using a Luminex 200 instrument (Luminex Corporation) and analyzed with StarStation2.3 software.

For flow cytometry analysis of innate cytokines, PBMCs were stimulated with indicated concentration of compound for 1 h followed by 5 h incubation with GolgiPlug (BD, 1 μL/mL) at 37°C. Post-stimulation, cells were harvested and surface stained with viability dye, and monoclonal antibodies targeting CD3 AF700 (Tonbo Bioscience, UCHT1), CD19 AF700 (Tonbo Bioscience, HIB19), CD56 AF700 (Biolegend, 5.1H11), HLA-DR BV785 (Biolegend, L243), CD14 APC-Cy7 (Biolegend, 63D3), CD16 PE-Dazzle594 (Biolegend, 3G8), CD11c BV421 (Biolegend, 3.9), and CD123 APC (Biolegend, 6H6). Following fixation and permeabilization with BD Cytofix/Cytoperm buffers, cells were stained with monoclonal antibodies targeting the intracellular cytokines IL-12p40 PE (a shared IL-12 and IL-23 subunit, Biolegend, C11.5), IL-6 FITC (Biolegend MQ2-13A5), IL-4 BV605 (Biolegend, MP4-25D2), and LAP (TGF-β) PE Cy7 [latency-associated peptide (55), Biolegend TW4-2F8]. Data were collected using an LSRII flow cytometer (BD) and analyzed using FlowJo 10.0 software (TreeStar).

### *In vivo* Experiments

Animal studies were carried out in accordance with University of Montana's IACUC guidelines for the care and use of laboratory animals. Groups of 6 female BALB/c mice were vaccinated intramuscularly with 0.3 μg HA equivalent monovalent detergent-split A/Victoria/210/2009 (H3N2; monovalent detergent split influenza vaccine was provided by GSK Vaccines) influenza vaccine with or without the indicated concentrations of TLR agonists in 50 μL total volume per injection (compounds and antigen were diluted as needed in 2% glycerol in water). After 14 days, blood samples were collected via submandibular bleeds for antibody analysis and a secondary vaccination was administered. At day 19 (5 days post-secondary vaccination), mice were euthanized and spleens were harvested for the assessment of cell-mediated immunity. For influenza challenge experiments, 16 female Balb/c mice per group were vaccinated intramuscularly with 0.3 μg detergent-split A/Victoria influenza vaccine with or without the indicated concentrations of TLR agonists in 50 μL total volume per injection. After 14 days, blood samples were collected for antibody analysis and a secondary vaccination was administered. At day 19 (5 days post-secondary vaccination), 6 mice per group were euthanized and spleens were harvested to assess T cell responses. Fourteen days after secondary vaccination, blood was collected from remaining 10 mice via submandibular bleeds for serum antibody analysis. Three weeks following the secondary vaccination, the remaining 10 mice in each group were anesthetized with approximately 10 mg/kg ketamine/xylazine i.p. and challenged with 10 μL per nare with mouse-adapted A/Hong Kong/1/68 (H3N2) at a dose of 3LD_50_. Clinical body scores, temperatures, body weights and mortality were recorded daily for each mouse. The humane endpoint for euthanasia included any of the following: (1) 30% weight loss, (2) body temperature <25°C two consecutive days, or (3) clinical score of 4.

### ELISA for Anti-influenza Antibody Quantification

Blood was collected from mice 14 days post-primary, serum was isolated and diluted according to the expected antibody response (between 1:10 and 1:5000). Plates were coated with 100 μL of detergent-split A/Victoria influenza vaccine at 1 μg/mL. Following washing (PBS plus tween 20) and blocking (SuperBlock, Scytek Laboratories), plates were incubated with diluted serum for 1 hr followed by anti-mouse IgG, IgG1 or IgG2a-HRP secondary antibody (Bethyl Laboratories) and TMB substrate (BD). Plates were read at 450 nm. Antibody titers were determined by calculating titer of each sample at OD 0.3.

### Splenocyte Restimulation and Cell-Mediated Immunity Analysis

Spleens were harvested from vaccinated mice 5 days after secondary injections and processed cells by disruption of the spleens through a 100 μm filter. Red blood cells were lysed by incubation with red blood cell lysis buffer (Sigma) for 5 min followed by washing in 1x PBS. Cells were plated in a 96 well-plate at 5 × 10^6^ cells/well in 200 μL complete RPMI1640 media. Cells were incubated with 1 μg/mL whole influenza antigen (detergent split A/Victoria or whole HK68 as indicated) plus 1 μg/mL αCD28 and 1 μg/mL αCD49d for 6 h at 37°C. After 6 h, 1 μL/mL GolgiPlug (Brefeldin A, BD Biosciences) was added to each well and cells were incubated at 37°C for a further 12 h. Following incubation, cells were stained with the cell surface antibodies against CD3e PerCP-Cy5.5 (Tonbo Biosciences, 145-2C11), CD4a APC-Cy7 (Tonbo Biosciences, RM4-5) and CD8a PE-Cy7 (Tonbo Biosciences, 53-6.7) and viability stain (Ghost 510, Tonbo Biosciences). Cells were treated with Cytofix/Cytoperm (BD) and stained with anti-IFNγ PE-CF594 (BD, XMG1.2), anti-IL2 FITC (Biolegend, JES6-5H4), anti-IL-5 BV421 (BD, TRFK5), anti-IL17A PE (Biolegend, TC11-18H10.1), and anti-TNFα APC (Invitrogen, MP6-XT22). Data was collected using an LSRII flow cytometer (BD) and analyzed using FlowJo 10.0 software (TreeStar).

Secreted cytokines following spleen harvest and antigen restimulation (1 μg/mL whole influenza antigen, detergent split A/Victoria or whole HK68 as indicated) were measured after 72 h of stimulation by MesoScale Discovery (MSD) U-PLEX Assay Platform (MesoScale Diagnostics) to detect mouse IFNγ, IL-17, TNFα, IL-2, and IL-5.

## Results

### Structure and Activity of Novel Lipidated TLR7/8 Agonists

**Figure 1:**
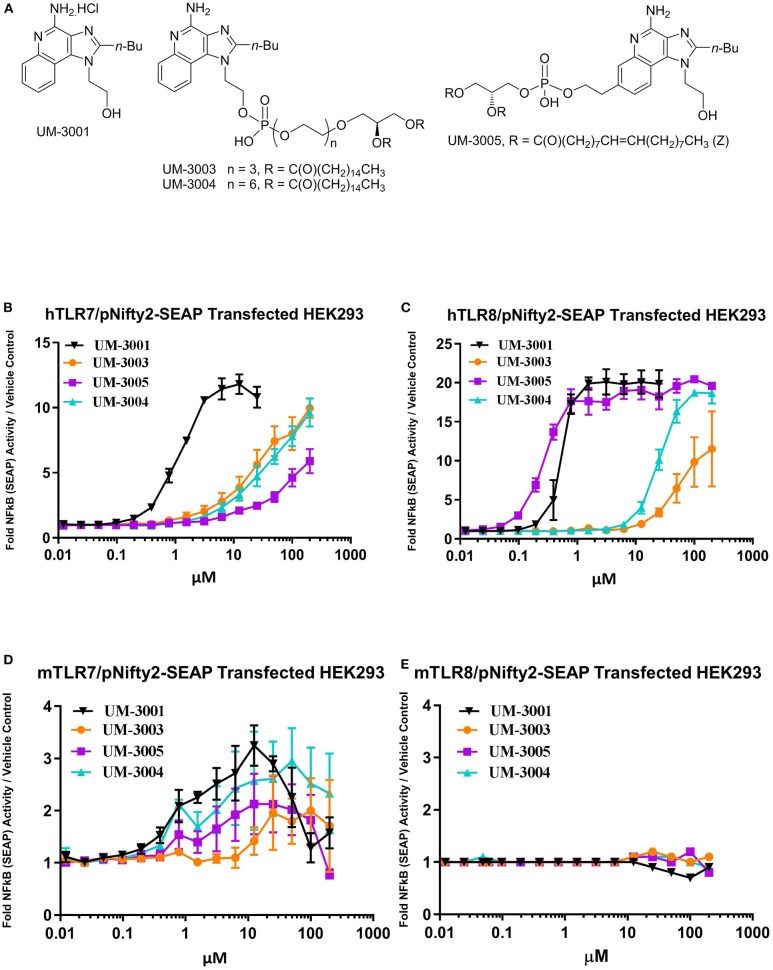
Novel imidazoquinolines as TLR7/8 agonists. **(A)** Chemical structures of synthesized imidazoquinoline small molecules. **(B,C)** Activity of imidazoquinolines in human embryonic kidney (HEK) reporter cells expressing human TLR7 (**B**; *n* = 2) or human TLR8 (**C**; *n* = 2). **(D,E)** Activity of imidazoquinolines in human embryonic kidney (HEK) reporter cells expressing mouse TLR7 (**D**; *n* = 3) or mouse TLR8 (**E**, *n* = 3). In b-e, cells were stimulated with imidazoquinolines formulated in 2% glycerol at the indicated concentrations for 24 h. HEK reporter activity is expressed as fold change over media only control.

Compounds shown in Figure 1A were formulated in 2% glycerol and tested in human and mouse TLR7 and TLR8 HEK293-SEAP reporter cells to determine their relative TLR7 and TLR8 receptor specificity and potency. Briefly, the HEK reporter system consists of HEK293 cells that express either human or mouse TLR7 or TLR8. When signaling occurs through the expressed TLR resulting in activation of NFkB, a SEAP reporter is expressed and measured via a colorimetric assay using cell culture supernatants (Figures 1B,C). UM-3001 (non-lipidated) demonstrated strong NFkB activation through both human TLR8 (EC50 = 0.53 μM, Figure 1C and TLR7 (EC50 = 1.12 μM, Figure 1B) while UM-3005 (lipidated at the 7-position) elicited strong NFkB activation through human TLR8 (EC50 = 0.27 μM, Figure 1C) but was much less potent with respect to NFkB activation via human TLR7 (EC50 = 499.2 μM, Figure 1B). Moving the phospholipid from the 7-position to the 2-position of the core imidazoquinoline compound altered the TLR7/8 signaling as demonstrated for compounds UM-3004 and UM-3003, both lipidated at the 2-position. UM-3004 induced signaling through NFkB via both TLR7 (EC50 = 60.5 μM; Figure 1B) and TLR8 (EC50 = 24.6 μM; Figure 1C). UM-3003 behaved similarly to UM-3004, signaling through NFkB via TLR7 (EC50 = 34.7 μM; Figure 1B) and minimally via TLR8 (EC50 = 52.5 μM; Figure 1C). As has been previously reported (56), mouse TLR8 is not readily activated by imidazoquinolines and other TLR8 ligands [Figure 1E; R848 plus polyDT was included as a positive control for mouse TLR8 activity as reported in (56) shown in Figure S1], despite their ability to signal through human TLR8 (Figure 1C). However, all compounds activated mouse TLR7 as demonstrated in Figure 1D. UM-3001, UM-3004, and UM-3005 were more potent activators of mouse TLR7 than UM-3003, as demonstrated by lower EC50s (UM-3001 = 0.48 μM, UM-3004 = 0.86 μM, UM-3005 = 1.14 μM, UM-3003 = 12.64 μM) in the HEK293 assay system (Figure 1D). None of the compounds shown in Figure 1A elicited signaling via NFkB when tested in the HEK Null line containing the NFkB-SEAP reporter without human or murine TLR7/8 (Figure S1). CRX-601 has been previously published and validated as a TLR4 agonist (57).

### TLR7/8 Adjuvanted Influenza Vaccination Elicits a Th1/Th17-Biased Immune Response in Mice

**Figure 2:**
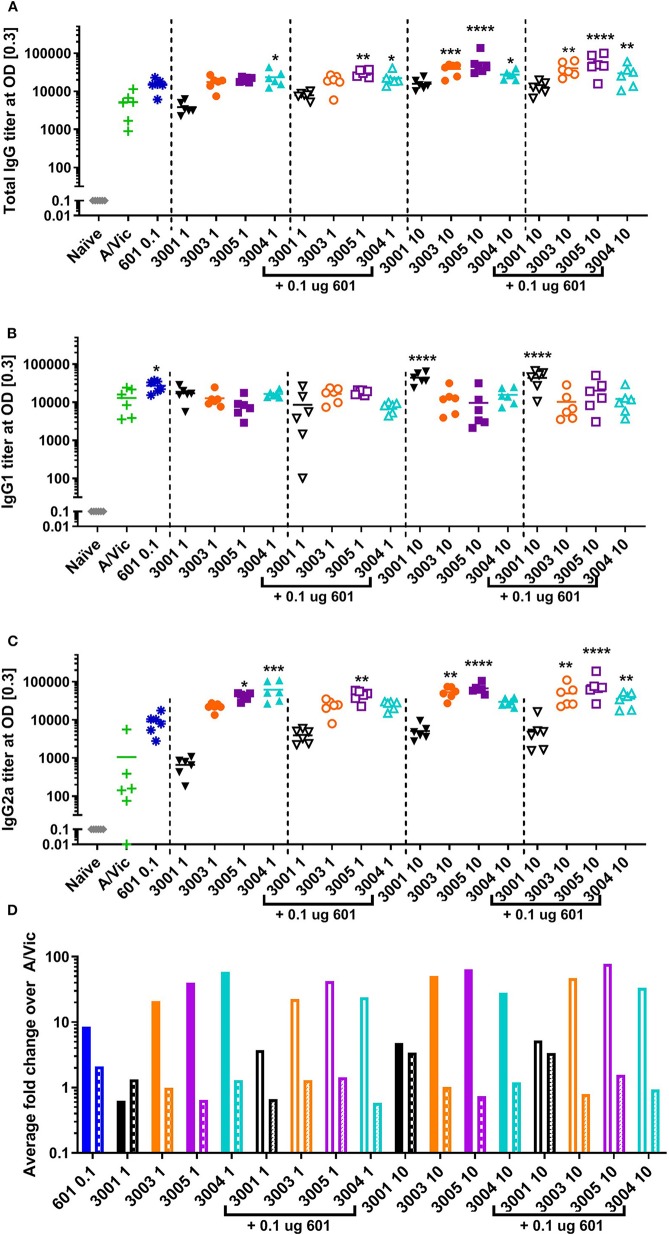
Lipidated imidazoquinolines elicit an IgG2a-biased influenza-specific antibody response after a single intramuscular vaccination. Balb/c mice (6 per group) were injected once i.m. with 0.3 μg/mouse A/Vic monovalent detergent-split influenza vaccine adjuvanted with indicated TLR4 (0.1 μg), TLR7/8 (1 or 10 μg as indicated), or combination TLR4 + TLR7/8 agonists as indicated. Fourteen days post-injection, mice were bled, serum was collected, and A/Vic-specific total IgG **(A)**, IgG1 **(B)** and IgG2a **(C)** antibody titers were measured. **(D)** Fold change of IgG2a (solid bars) or IgG1 (patterned bars) titers with indicated adjuvant normalized to non-adjuvanted (A/Vic alone) control. Statistical signficance determined by one-way ANOVA (GraphPad Prism 7) followed by Fishers LSD for multiple comparisons; asterisks indicate significance compared to the A/Vic alone group (green “+”) where *p ≤ 0.05, **p ≤ 0.01, ***p ≤ 0.001, ****p ≤ 0.0001.

Previous success using lipidated TLR7/8 agonists by other groups, namely 3M-052 (43) and 1V270 (58), led us to hypothesize that these novel imidazoquinolines may serve as potent adjuvants for influenza vaccination in mice. Additionally, we hypothesized that the addition of a synthetic TLR4 agonist may further enhance the adaptive immune responses as previous reports have noted synergy between TLR4 agonists and TLR7/8 agonists, especially with respect to the generation of a Th1 type immune response (45–49, 58). To investigate these hypotheses, a monovalent detergent-split A/Victoria/210/2009 (H3N2; A/Vic) influenza vaccine (0.3 μg/mouse) was adjuvanted with either low dose TLR7/8 agonist (1 μg/mouse) with or without 0.1 μg CRX-601 (1:10 ratio of CRX-601:TLR7/8 agonist), or high dose TLR7/8 agonist (10 μg/mouse) with or without 0.1 μg CRX-601 [1:100 ratio of CRX-601:TLR7/8 agonist; (56]]. Two injections were administered intramuscularly (i.m.) 14 days apart. Mice were bled 14 days post-primary injection (14pd1) to measure influenza-specific antibody responses and spleens were harvested 5 days post-secondary injection (5dp2) to assess influenza-specific T cell responses. Total influenza-specific IgG serum titers were increased by high-dose lipidated adjuvants (UM-3003, UM-3005, and UM-3004) alone or in combination with 601 (Figure 2A). Low dose UM-3004 alone and low dose UM-3004 and UM-3005 plus 601 also significantly increased total influenza-specific IgG serum titers. Influenza-specific IgG1 was significantly increased only by 601 alone, high dose UM-3001 (non-lipidated TLR7/8 adjuvant), and high dose UM-3001 with 601 (Figure 2B). Conversely, influenza-specific IgG2a was significantly increased in the majority of groups that contained a lipidated TLR7/8 agonist (Figure 2C): all groups adjuvanted with UM-3005, all groups adjuvanted with high dose lipidated TLR7/8 agonist plus 601, as well as high dose UM-3003 alone, and low dose UM-3004 alone. The ability of lipidated TLR7/8 agonists to drive an IgG2a response compared to the ability of non-lipidated TLR7/8 agonists to drive an IgG1 response is illustrated in Figure 2D, where the average fold change of adjuvanted influenza-specific IgG1 or IgG2a titers over A/Vic alone (no adjuvant) was calculated (Figure 2D; solid or open bars indicate IgG2a fold change, patterned bars indicate IgG1 fold change). This calculation allows for a direct comparison between the change in IgG2a vs. IgG1 antibody titers in mice vaccinated with adjuvant plus antigen compared to mice vaccinated with antigen alone, demonstrating whether an adjuvant drives IgG2a or IgG1 antibody production. Lipidated TLR7/8 adjuvants drove a predominantly IgG2a influenza-specific response compared to non-adjuvanted A/Vic (Figure 2D) as evidenced by the fact that IgG2a titers were increased more than IgG1 titers by adjuvanting with UM-3003, -3004, and -3005 compared to A/Vic alone. The non-lipidated TLR7/8 adjuvant, UM-3001, produced an approximately equal increase in both IgG2a and IgG1 titers compared to A/Vic alone (Figure 2D).

**Figure 3:**
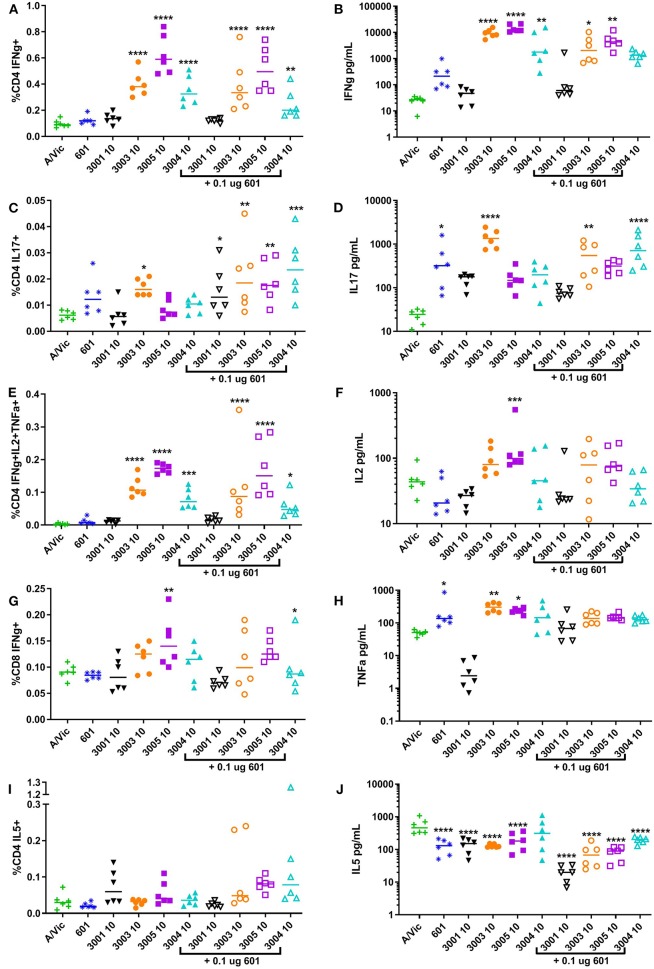
High-dose lipidated imidazoquinolines elicit an influenza-specific Th1 response and, in combination with a TLR4 agonist, an influenza-specific Th17 response. Balb/c mice (6 per group) were injected twice i.m. fourteen days apart with 0.3 μg/mouse A/Vic monovalent detergent-split influenza vaccine adjuvanted with indicated TLR4 (0.1 μg/mouse), TLR7/8 (10 μg/mouse), or combination TLR4 + TLR7/8 agonists. Five days post-secondary injection (5dp2), mice were euthanized and spleens were harvested, disaggregated and restimulated with 1 μg/mL A/Vic + 1 μg/mL αCD28 + 1 μg/mL αCD49d for flow cytometry or 1 μg/mL A/Vic alone for MSD. The indicated cytokines were measured by flow cytometry cytokines after 6 h of stimulation plus a further 12 h with GolgiPlug (BD Biosciences) followed by intracellular cytokine staining (left column; **A**, **C**, **E**, **G**, **I**). Secreted cytokines were measured via MesoScale Discovery (MSD) assay after 72 h of stimulation (right column; **B**, **D**, **F**, **H**, **J**). Lines indicate means. Statistical signficance determined by one-way ANOVA followed by Fishers LSD for multiple comparisons (GraphPad Prism 7); asterisks indicate significance compared to the A/Vic alone group (green “+”) where *p ≤ 0.05, **p ≤ 0.01, ***p ≤ 0.001, ****p ≤ 0.0001.

Influenza-specific T cell responses were measured in splenocytes harvested at 5 days post-secondary vaccination and re-stimulated *ex vivo* with A/Vic (H3N2) split flu antigen, followed by flow cytometry to detect intracellular cytokines and MesoScale Discovery (MSD) multiplex cytokine array to detect secreted cytokines. Here, we used the A/Vic only vaccinated mice as a control as opposed to the more commonly seen method of using unstimulated splenocytes as a control, allowing the changes in the immune response that are directly due to the adjuvant to be determined. Mice vaccinated with A/Vic plus non-lipidated TLR7/8 (UM-3001) did not exhibit any significantly increased cytokine production compared to mice vaccinated with A/Vic alone (Figure 3: high dose adjuvant T cell responses; Figure S3: low dose adjuvant T cell responses). In contrast, mice vaccinated with lipidated TLR7/8 agonists UM-3003, UM-3005 or UM-3004 exhibited primarily a Th1 influenza-specific response in mice (Figures 3A,B; flow gating and example cytokine staining shown in Figure S2; low dose adjuvant T cell responses shown in Figure S3) which correlates with the IgG2a biased antibody responses also elicited by these adjuvants (see Figure 2). High dose lipidated TLR7/8 agonists, with or without the addition of a TLR4 agonist, also elicited significantly increased frequencies of influenza-specific multifunctional CD4 T cells (IFNγ+ IL2+ TNFα+, Figure 3B; individual secreted cytokines shown in Figures 3B,F,H), which have previously been shown to be beneficial for protection against heterologous influenza challenge (59). Frequencies CD8 IFNγ+ cells, of particular interest when trying to elicit an anti-viral response, were significantly increased through adjuvanting A/Vic antigen with high dose UM-3005 with or without a TLR4 agonist (Figure 3G). Interestingly, the addition of a TLR4 agonist did not boost influenza-specific Th1 responses but did significantly increase influenza-specific Th17 responses when combined with high dose lipidated TLR7/8 agonists (Figures 3C,D). Splenocytes from vaccinated mice adjuvanted with only CRX-601 also demonstrate IL-17 secretion after *ex vivo* antigen restimulation (Figure 3D). None of the adjuvants produced significant increases in CD4 IL-5+ frequencies or in concentration of secreted IL-5; in fact, cytokine secretion as measured by MSD indicates that all adjuvants, except high dose UM-3004 alone, reduced IL-5 secretion compared to non-adjuvanted A/Vic antigen (Figure 3J, Figure S3J). These T cell data are in agreement with antibody data, demonstrating that lipidated TLR7/8 agonists biased a Th1 response without boosting the Th2 type response whereas the non-lipidated TLR7/8 agonist did not elicit any measurable antigen-specific T cell responses. T cell data also demonstrates that very low dose TLR4 agonist elicited a low level Th17 response and, when added to lipidated TLR7/8 agonists, increased their ability to elicit a Th17 response as well.

### TLR7/8 Adjuvanted Influenza Vaccine Protects Against Heterologous H3N2 Influenza Challenge in Mice

**Figure 4:**
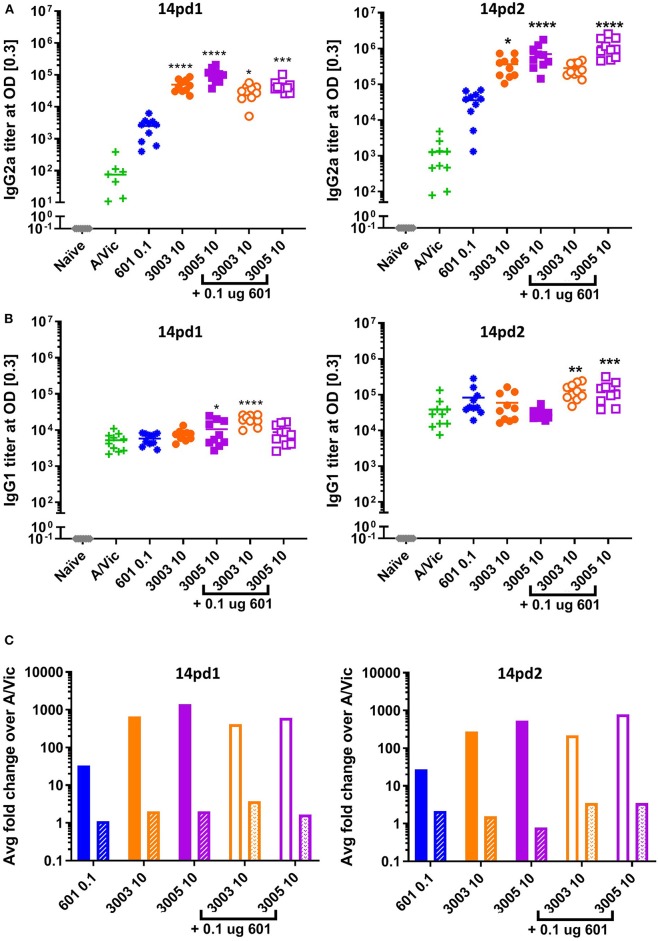
Th1 humoral bias is maintained after two vaccinations. Balb/c mice (10 per group) were vaccinated i.m. with A/Vic detergent-split monovalent influenza vaccine (0.3 μg/mouse) with indicated adjuvants (CRX-601 = 0.1 μg/mouse, UM-3003 or UM-3005 = 10 μg/mouse). At 14dp1 and 14dp2 mice were bled and influenza-specific IgG2a **(A)** and IgG1 **(B)** titers were measured. **(C)** Fold change of adjuvanted IgG2a (solid bars) or IgG1 (patterned bars) compared to non-adjuvanted control (A/Vic only) at 14dp1 (left) and 14dp2 (right). Lines indicate mean. Statistical signficance determined by one-way ANOVA followed by Fishers LSD for multiple comparisons (GraphPad Prism 7); asterisks indicate significance compared to the A/Vic alone group (green “+”) where *p ≤ 0.05, **p ≤ 0.01, ***p ≤ 0.001, ****p ≤ 0.0001.

**Figure 5:**
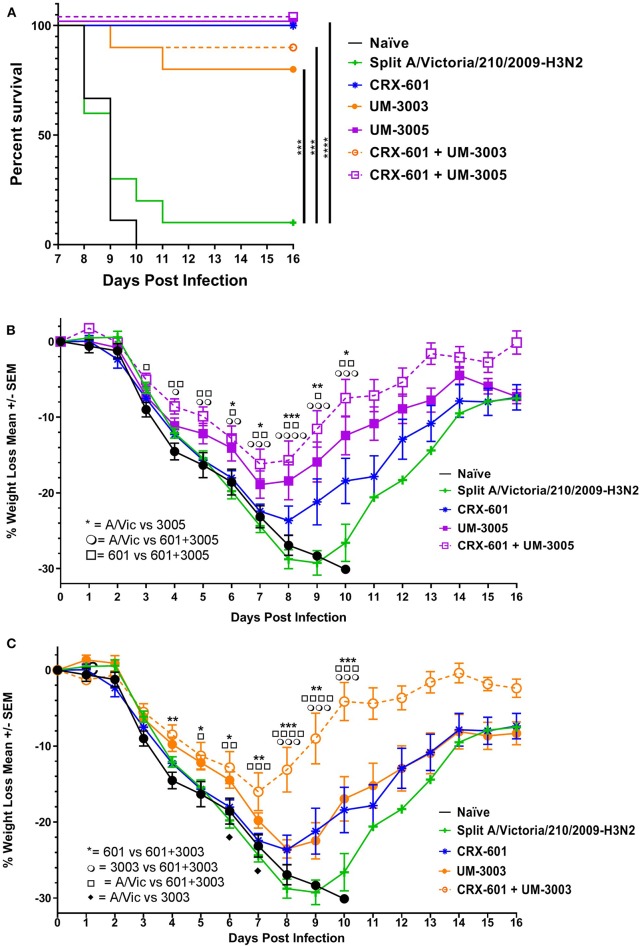
Lipidated imidazoquinolines with or without a TLR4 agonist protect against lethal, heterologous H3N2 challenge. Balb/c mice were immunized i.m. twice, 14 days apart, with A/Vic monovalent detergent-split influenza vaccine (0.3 μg/mouse) adjuvanted with indicated TLR4 (0.1 μg/mouse), TLR7/8 (10 μg/mouse), or TLR4 + TLR7/8 agonists. Three weeks after the second immunization, 10 mice per group were challenged i.n. with 3LD50 HK68 (H3N2) mouse-adapted influenza virus. Mice were monitored daily for weight loss, temperature, and body condition. **(A)** Percent of surviving mice in each group. **(B,C)** Average percent weight loss per group, +/- SEM. Survival statistics determined by log-rank (Mantel-Cox) test, weight loss significance determined by one-way ANOVA at a given timepoint followed by Fishers LSD for multiple comparisons (GraphPad Prism 7); *p ≤ 0.05, **p ≤ 0.01, ***p ≤ 0.001, ****p ≤ 0.0001. For ease of comparison, data for UM-3005 and CRX-601 + UM-3005 adjuvanted mice are displayed in **(B)** along with control groups (CRX-601 adjuvanted mice, A/Vic vaccinated mice, and naïve mice). Similarly, data for UM-3003 and CRX-601 + UM-3003 are displayed in **(C)** along with control groups (CRX-601 adjuvanted mice, A/Vic vaccinated mice, and naïve mice).

Based on promising vaccine-induced influenza-specific antibody and T cell responses, we next evaluated whether or not vaccinated mice are protected against a heterologous H3N2 influenza virus challenge. For this purpose, we used a mouse-adapted A/Hong Kong/1/68 (HK68) challenge virus. Groups of 16 mice were vaccinated as above with A/Vic antigen alone or A/Vic antigen adjuvanted with TLR4 agonist (CRX-601, 0.1 μg/mouse), TLR7/8 agonist (UM-3003 10 μg/mouse or UM-3005 10 μg/mouse), or combination TLR4 agonist plus TLR7/8 agonist (0.1 μg/mouse TLR4 + 10 μg/mouse TLR7/8; 1:100 ratio). Lead compounds, combinations, ratios and doses were selected based on previous results (see above, Figures 2, 3). UM-3005 is structurally unique compared to UM-3003 and -3004 and elicited the strongest Th1-biased T cell and antibody responses at 10 μg, either alone or in combination with CRX-601. We also elected to continue experiments with 10 μg UM-3003 alone and in combination with CRX-601. Due to UM-3004's structural similarity to UM-3003 and similar but slightly less promising immune profile, this compound was not carried forward into challenge studies. Influenza-specific antibody titers (14dp1) and T cell responses (5dp2) were assessed to confirm that immune responses as described above (Figures 2, 3) were replicated. Influenza-specific IgG2a (Figure 4A, left), IgG1 (Figure 4B, left) and T cell responses (Figures S4A–H) were very similar to those observed in the adjuvant response study (Figures 2, 3, Figures S2, S3) with one exception: UM-3003 plus A/Vic antigen no longer elicited a significantly higher IL-17 response compared to A/Vic antigen alone (Figures S4E,F). However, all other responses were replicated both in terms of humoral response IgG2a bias (Figure 4C, left) and Th1 (Figures S4A–C) and Th17 (Figures S4E,F) CD4 T cell responses. In addition, serum antibody titers were measured 14 days following the secondary vaccination (14dp2; Figure 4, right column). Both IgG2a (Figure 4A, right) and IgG1 (Figure 4B, right) influenza-specific antibody titers continued to increase compared to 14dp1; particularly the group vaccinated with A/Vic with CRX-601 plus UM-3005 (Figures 4A,B). As demonstrated at 14dp1, at 14dp2 all adjuvanted groups compared to non-adjuvanted A/Vic alone increased average IgG2a titers to a greater degree than average IgG1 titers (Figure 4C, right), demonstrating that after a second injection, the Th1 humoral bias remains. All remaining mice (10 per group) were challenged with 3LD_50_ mouse-adapted A/Hong Kong/1/68 (H3N2; HK68) 21 days after the secondary vaccination via the intranasal/intrapulmonary route (10 μL/nare). As expected, all of the naïve (non-vaccinated) mice succumbed to the 3LD50 influenza virus challenge with 100% mortality by day 10 following challenge (Figure 5A). Mice vaccinated with A/Vic antigen (no adjuvant) demonstrated limited protection with 10% survival (1 mouse out of 10). All groups that received adjuvanted A/Vic antigen were protected when challenged with the heterologous mouse adapted A/HK/68 influenza virus (Figure 5A; protection is considered as 80% survival or greater). However, some groups experienced significantly greater weight loss indicating differences in the level of protection observed. Mice vaccinated with A/Vic antigen plus CRX-601 (a TLR4 ligand) experienced significantly greater weight loss compared to mice that received A/Vic antigen and CRX-601 in combination with UM-3005 or UM-3003 (Figures 5B,C; dark purple and dark orange lines, respectively). Weight loss in mice receiving antigen plus UM-3005 was somewhat greater than that experienced by mice adjuvanted with UM-3005 in combination with CRX-601 although this difference was not statistically significant (Figure 5B). Interestingly, weight loss experienced by mice vaccinated with antigen and CRX-601 plus UM-3005 vs. CRX-601 plus UM-3003 was not statistically different (Figures 5B,C), despite the fact that mice vaccinated with UM-3003 plus antigen experienced significantly greater weight loss than those receiving UM-3003 plus CRX-601 (Figure 5C). These data indicate that a combination adjuvant including a synthetic TLR4 agonist and a lipidated TLR7/8 agonist induces better protection against influenza-induced weight loss than a TLR4 adjuvant or a TLR7/8 agonist alone (Figure 5C).

### TLR7/8 Stimulation of PBMCs Elicits a Th1/Th17 Polarizing Innate Cytokine Response

**Figure 6:**
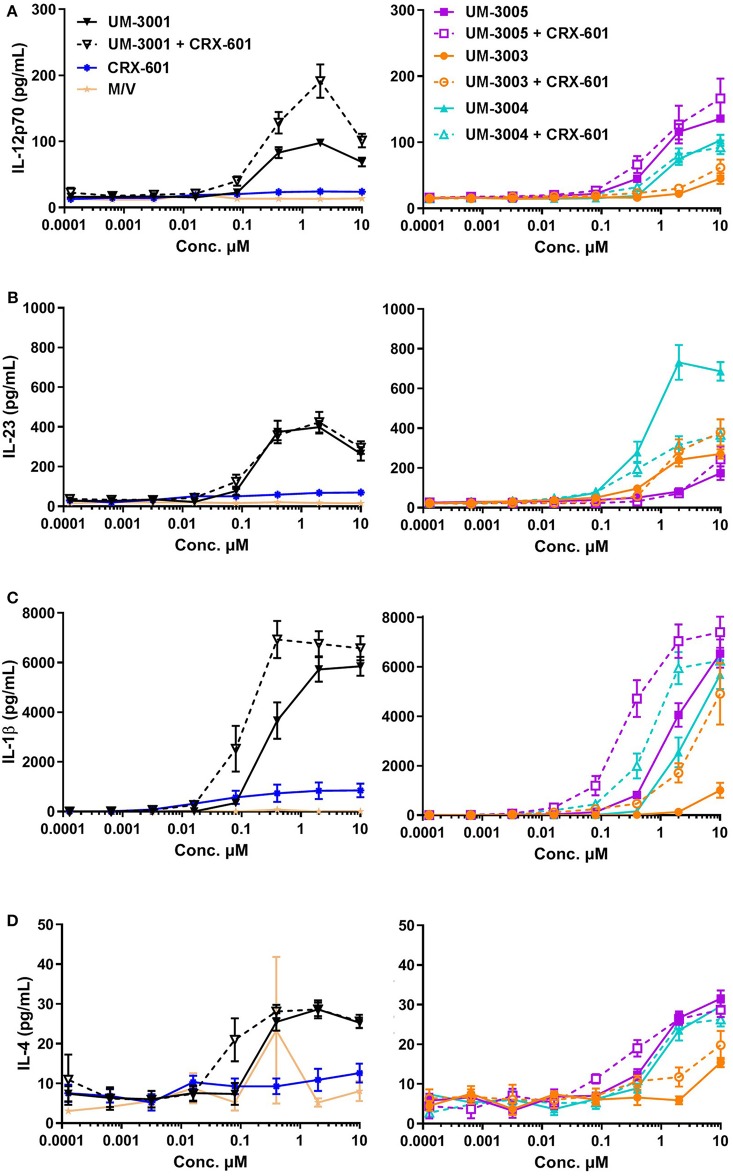
Lipidated imidazoquinolines elicit a Th1/Th17-inducing innate cytokine profile from human PBMCs. PBMCs from 6 healthy, adult blood donors were stimulated with indicated concentrations of TLR7/8 agonists with and without a TLR4 agonist. Secreted cytokines were measured after 24 h via Luminex assay. Supernanant concentrations of **(A)** IL-12p70, **(B)** IL-23, **(C)** IL-1β, and **(D)** IL-4 were determined. For clarity, cytokine secretion data from PBMCs stimulated with media only (M/V), CRX-601, non-lipidated TLR7/8 agonist UM-3001, and UM-3001+CRX-601 are displayed in left plots while cytokine secretion data from PBMCs stimulated with lipidated TLR7/8 agonists alone and in combination with CRX-601 are diplayed on right plots. Note that for each cytokine measured, the y-axis scale used for the left and right plots is identical for ease of comparison.

To determine if the lipidated TLR7/8 ligands may be capable of eliciting a similar T cell response in humans, we investigated production of T cell polarizing cytokines in human PBMCs both by flow cytometry and via R&D Systems multiplex cytokine array (Luminex). In addition to evaluating the TLR7/8 ligands alone, we also evaluated potential synergies between CRX-601 and the TLR7/8 ligands. IL-12p70, the canonical Th1-polarizing cytokine (60–62), is produced to various degrees by all four TLR7/8 agonists, both lipidated and non-lipidated (Figure 6A), although UM-3003 only produces low levels of IL-12p70 at the highest tested concentration (Figure 6A, right). As previously reported for other TLR7/8 agonists *in vitro* (44–46], the addition of a TLR4 ligand CRX-601 at a 1:10 ratio boosted IL-12p70 production when combined with the non-lipidated TLR7/8 ligand UM-3001 (Figure 6A, left) while CRX-601 stimulation alone did not elicit a detectable IL-12p70 response (Figure 6A, left). IL-23 and IL-1β are important for Th17 polarization (63). This combination of cytokines was also produced to various degrees by all four TLR7/8 agonists investigated here (Figures 6B–D), again with UM-3003 eliciting low concentrations of IL-23 and IL-1β at the highest tested dose (Figures 6B,C, right). Interestingly, the addition of CRX-601 strongly enhanced IL-1β production despite eliciting low levels of IL-1β by itself (Figure 6C, left), suggesting there may be a synergistic IL-1β response to TLR4 plus TLR7/8 stimulation. IL-6, also shown to contribute to Th17 polarization (citation as above, 61) was also elicited by all adjuvants, including CRX-601 alone (Figure S5A). IL-4 production, which polarizes Th2 cells, was found to be secreted only at very low concentrations by these TLR7/8 agonists (Figure 6G). Additionally, IFNα, an important cytokine for protection against viral infections and Th1 polarization [reviewed in (64)], is secreted in response to lipidated imidazoquinolines UM-3003, UM-3005, and UM-3004 but not by the non-lipidated TLR7/8 ligand UM-3001 (Figure S5B) despite its potent TLR7 activity in the HEK293 reporter assay (see Figure 1B).

**Figure 7:**
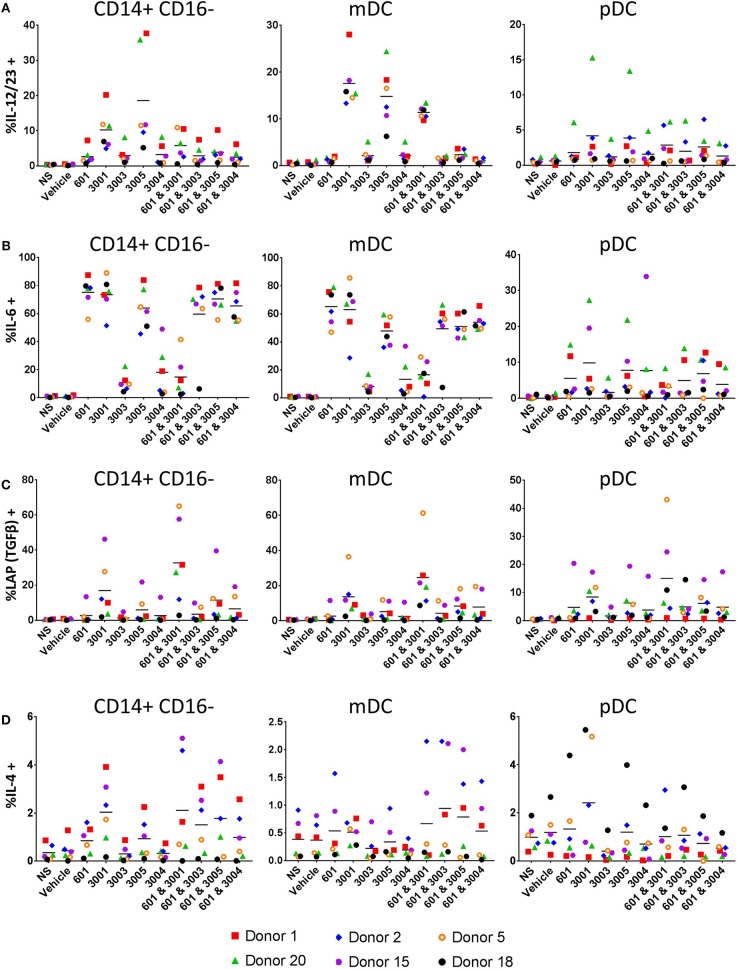
Lipidated imidazoquinolines elicit T cell polarizing cytokines primarily from APCs. PBMCs from 6 healthy, adult blood donors were stimulated with 2 μM indicated TLR7/8, TLR4, or combination TLR4 + TLR7/8 agonists. After 1 h, brefeldin A (GolgiPlug) was added and cells were incubated for a further 5 h. Cells were then harvested and stained with surface antibodies for phenotyping, fixed and permeablized and stained with antibodies to detect intracellular cytokines. **(A)** Frequency of IL-12/IL-23+ cells, **(B)** frequency of IL-6+ cells, **(C)** frequency of TGFβ (LAP)+ cells, and **(D)** frequency of IL-4+ cells.

IL-12p40, the shared subunit of IL-12 and IL-23, is produced mainly by myeloid dendritic cells (mDCs) and monocytes in response to both UM-3005 and UM-3001 (Figure 7A, left (mDCs) and right (monocytes)). Lower frequencies of mDC and classical monocytes produce IL-12p40 in response to the weaker TLR7/8 agonists (UM-3004) and the weak TLR7 agonist (UM-3003) (Figure 7A). Interestingly, the addition of a TLR4 agonist reduced frequencies of IL-12p40+ mDC and monocytes (Figure 7A). Additionally, approximately 20% of mDC and classical monocytes produce IL-12p40 (the shared IL-12 and IL-23 subunit) in response to UM-3005 and, in the case of mDC, UM-3001 (Figure 4B). Interestingly, TLR4 stimulation induces a high frequency of mDC (median 60%) and classical monocytes (median 80%) to produce IL-6 (Figure 7B). In combination with weak TLR7/8 stimulation (UM-3004 and UM-3003), frequency of IL-6 production is somewhat lowered compared to TLR4 stimulation alone but when in combination with a strong TLR7/8 agonist (UM-3001), the frequency of IL-6 producing mDC or classical monocytes is lower than of either alone (Figure 7B), suggesting either activation-induced cell death or that excessive activation is inducing negative regulators that serve to terminate signaling. TGFβ [as measured by latency-associated peptide, LAP (55), an intracellular immature form of TGFβ] is expressed by low frequencies of mDC and classical monocytes in response to CRX-601 plus UM-3001 and UM-3001 alone (Figure 7C). Interestingly, TGFβ/LAP production appears to have higher donor-to-donor variability compared to other cytokines measured here. Only very low frequencies of mDC, or classical monocytes (<6%) were found to express IL-4 in response to any of the TLR agonists investigated here (Figure 7D). In contrast to mDC and monocytes, pro-inflammatory/T cell polarizing pDC responses with respect to TLR7/8 stimulation were of lower frequency, as expected based on the expression of TLR7 but not TLR8 in pDCs (65) (Figure 7, right column). Data presented here confirm that the cytokine secretion as measured in Figure 6 comes from professional antigen-presenting cells that are best suited to present antigen to T cells and induce their polarization and differentiation. Further, secreted cytokine data shown in Figure 6 and the intracellular cytokine staining data (Figure 7) support the human HEK activity data shown in Figure 1B–UM-3005, the most strongly TLR8-biased compound, elicits the highest concentrations and frequencies of pro-inflammatory and Th1/Th17 biasing cytokines while UM-3003, the least TLR8-biased compound, elicits low concentrations and low frequencies of pro-inflammatory and Th1/Th17 polarizing cytokines but is the most potent inducer of IFNα, indicative of TLR7 activity. Taken together, the combination of cytokines expressed by mDC and classical monocytes, as well as secreted cytokine data, suggests a Th1 and Th17 polarizing innate cytokine environment upon stimulation with these TLR7/8 agonists in human PBMCs, particularly by UM-3005. Interestingly, UM-3005 was also the most potent single adjuvant with respect to polarizing an influenza-specific Th1 immune response in mice as well as protection against influenza virus challenge in vaccinated mice.

## Discussion

Here, we explored the ability of novel lipidated imidazoquinolines (TLR7/8 agonists) to act as adjuvants to confer protection against drifted H3N2 influenza both with and without a TLR4 agonist in mice. We found that these compounds elicited a Th1/Th17 type T cell response as well as a strongly Th1-biased humoral response. When used as adjuvants in combination with low dose A/Vic, these compounds were protective against the pandemic H3N2 strain HK68. When combined with a low dose of TLR4 agonist at a 1:100 ratio, mice were still protected from challenge with HK68 and weight loss was reduced when 601+UM-3003 was used as an adjuvant compared to UM-3003 alone. No vaccine-induced reactogenicity (as determined by visual inspection of the mice, e.g., ruffled fur, hunched posture, reluctance to move, or visible weight loss) was observed in any mouse at any time. Given the dissimilarity of TLR7 and TLR8 in mice compared to humans, we also evaluated the cytokine profile elicited by these compounds in human PBMCs. Promisingly, we found that cytokines required to generate a Th1 and Th17 response were produced by PBMCs in response to TLR7/8 agonists while they did not elicit cytokines required to bias a Th2 response. Although some donor-to-donor variability was observed in the frequency of cells producing a specific cytokine or the amount of cytokine that each donor's PBMCs secreted in response to adjuvant stimulation, all six donors responded to imidazoquinoline TLR7/8 stimulation with the same combination of cytokines. Taken together, these data indicate that imidazoquinolines lipidated at the 2-position or 7-position function as potent Th1/Th17 adjuvants in mice, protect against lethal influenza challenge, and elicit the cytokines required to generate the same Th1/Th17 response in humans.

Previous work has demonstrated the efficacy of using a TLR4 agonist in combination with a lipidated TLR7/8 agonist or a lipidated TLR7/8 agonist alone in protecting against influenza challenge (43, 58) either in a DMSO, liposome, or emulsion formulation. The work shown here extends these previous studies by demonstrating that lipidated imidazoquinolines in an aqueous formulation, with or without the addition of a TLR4 agonist, also serve as potent influenza adjuvants. The aqueous formulation used here, 2% glycerol, includes no excipients that may confound the adjuvant effect of the TLR7/8 or TLR4 compounds by inducing an immune response by itself, unlike emulsions or liposomes in which some of the immune response and protective effect was shown to be due to the emulsion or liposome formulation alone (43), demonstrating the adjuvant effects of the small molecule compounds themselves. Further, we found the potency of the adjuvants explored in this work may allow antigen dose sparing as we found protection was induced at an antigen dose of 0.3 μg HA per mouse after two injections whereas previous groups have used an antigen dose of 5 μg HA per mouse (58). In a pandemic setting, adjuvants that allow antigen dose sparing will be critical for allowing production of enough vaccine doses for mass vaccination.

H3N2 strains, regardless of reported antigenic mismatch, are more difficult to protect against than drifted H1N1 or influenza type B–a recent meta-analysis calculated that between 2004 and 2015, vaccine effectiveness against H3N2 strains was only 33% compared to 54% for influenza type B and 61–73% for H1N1 (1). Reasons for the low efficacy of H3N2 vaccination, especially against drifted strains, are still mostly unknown. A recent report demonstrated that egg-grown H3N2 vaccines lack a glycosylation site that is found in circulating H3N2 strains and that non-egg grown vaccines were able to induce higher neutralizing antibody titers against H3N2 viruses containing the glycosylation site compared to egg grown vaccines (66) which may partially explain poor vaccine-induced responses to circulating H3N2 viruses. In addition to moving toward cell-based methods of vaccine production, data presented in this manuscript demonstrate that adjuvanting with a lipidated imidazoquinoline in combination with a TLR4 agonist would likely provide broader protection against drifted H3N2 viruses.

Data suggest that while antibody responses, B cells, CD4 and CD8 T cells are all required to optimally clear an influenza infection protect against further infection (11, 67–75), transfer of influenza-specific CD4 T cells protects influenza-naïve mice from challenge in the absence of B cells or CD8 T cells (11). Further, T cell responses are critical for broad protection against different influenza viruses (73, 76–80). Here, we demonstrate that the small molecule adjuvants investigated induce significant influenza-specific IFNγ+ T cells and/or IL17 production compared to A/Vic alone. In the case of lipidated imidazoquinolines, with or without the addition of CRX-601, an influenza-specific Th1 humoral bias was also produced. Compared to A/Vic vaccination alone, all mice that received adjuvanted A/Vic had either significantly increased A/Vic-specific IFNγ responses or IL-17 responses and were protected from mortality upon challenge. Mice who received A/Vic adjuvanted with a combination of UM-3003 or UM-3005 plus CRX-601 demonstrated an IgG2a-biased humoral response and significantly increased A/Vic-specific IFNγ and IL-17 T cell responses, and were the best protected from weight loss in a heterologous challenge model. This suggests that in this model both a Th1-biased humoral response as well as Th1 and Th17 T cell responses are required for optimal protection against heterologous H3N2 infection.

Despite the differences between mouse and human TLR7/8, the cytokine profile elicited by the lipidated imidazoquinoline TLR7/8 agonists investigated in this manuscript is that which is required to elicit a Th1/Th17 biased T cell response. Further, we demonstrated that these T cell polarizing cytokines were produced by mDC and classical monocytes, cell types that are critical as APCs in an infection setting. Although most cytokines measured were not boosted through the addition of CRX-601, addition of 601 did boost IL-1β production compared to TLR7/8 agonists alone. This is particularly striking as IL-1β induces Th17 differentiation (81, 82) and IL-17+ T cell frequencies were boosted in mice *in vivo* with the addition of CRX-601 to the TLR7/8 agonists, further strengthening connection between *in vivo* mouse data and *ex vivo* human data. While our group has previously demonstrated that too much IL-17 production can be detrimental in influenza infection (57), it is likely that some Th17 cells are important for viral clearance as mice adjuvanted with CRX-601 + TLR7/8 agonist experienced reduced weight loss and quicker recovery than those adjuvanted with CRX-601 or TLR7/8 agonist alone. Also, previous reports have shown that transferred influenza-specific Th17 memory cells can protect naïve mice against influenza challenge (11). Further, TLR7/8 adjuvants, particularly those with a TLR8 bias, demonstrated robust activity in activating human infant APCs (28) and increased neonatal macaque pneumococcus immunogenicity *in vivo* (41). A TLR8 biased adjuvant therefore, such as UM-3005, may be particularly efficacious at increasing infant responses to influenza. Taken together, these data suggest that UM-3003 and UM-3005 when used in combination with CRX-601 vaccine adjuvants in a detergent split influenza vaccine may provide much needed cross-protection against heterologous strains of H3N2 in humans.

## Data Availability Statement

The datasets generated for this study are available on request to the corresponding author.

## Ethics Statement

The studies involving human participants were reviewed and approved by the University of Montana Institutional Review Board. The patients/participants provided their written informed consent to participate in this study. The animal study was reviewed and approved by the University of Montana Institutional Animal Care and Use Committee.

## Author Contributions

SM and JE designed experiments and analyzed data and wrote the manuscript draft. SM, VC, and MW performed experiments, data collection, and analysis. LB, ML, and HB synthesized compounds. LW and DB formulated compounds and provided physical characteristics. All authors discussed data, and reviewed and edited the final manuscript.

### Conflict of Interest

VC, MW, LB, ML, LW, DB, HB, and JE were employees of GlaxoSmithKline Vaccines when TLR7/8 compounds described here were first synthesized. The remaining author declares that the research was conducted in the absence of any commercial or financial relationships that could be construed as a potential conflict of interest.
