# Supplementary material for: Novel Lipidated Imidazoquinoline TLR7/8 Adjuvants Elicit Influenza-Specific Th1 Immune Responses and Protect Against Heterologous H3N2 Influenza Challenge in Mice
Source: Front Immunol. 2020 Mar 10;11:406. doi: 10.3389/fimmu.2020.00406 (PMC7075946; doi:10.3389/fimmu.2020.00406)
Supplement: Supplementary file 1 [file Presentation_1.PPTX]

## Slide 1
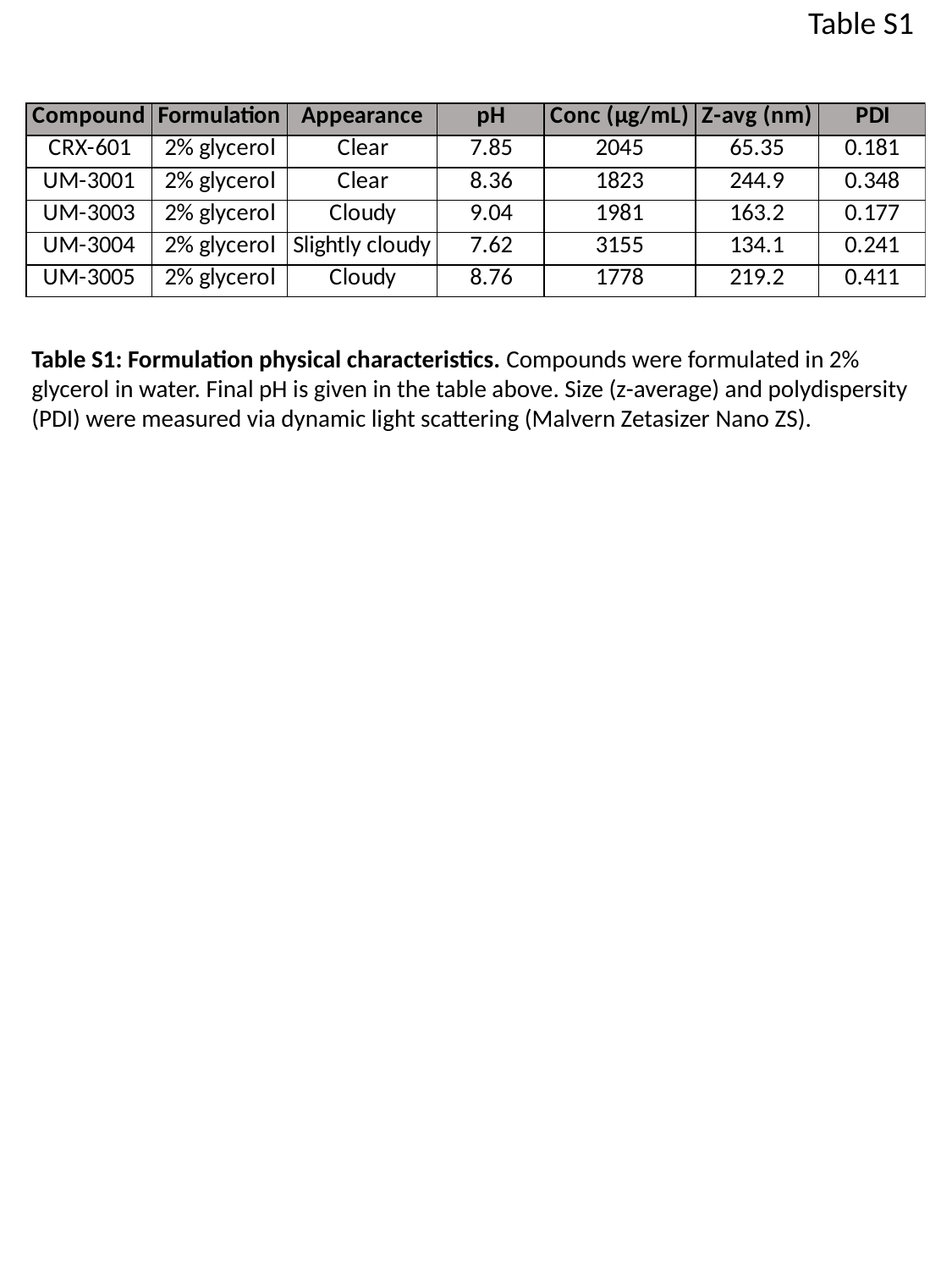

Table S1
Table S1: Formulation physical characteristics. Compounds were formulated in 2% glycerol in water. Final pH is given in the table above. Size (z-average) and polydispersity (PDI) were measured via dynamic light scattering (Malvern Zetasizer Nano ZS).

## Slide 2
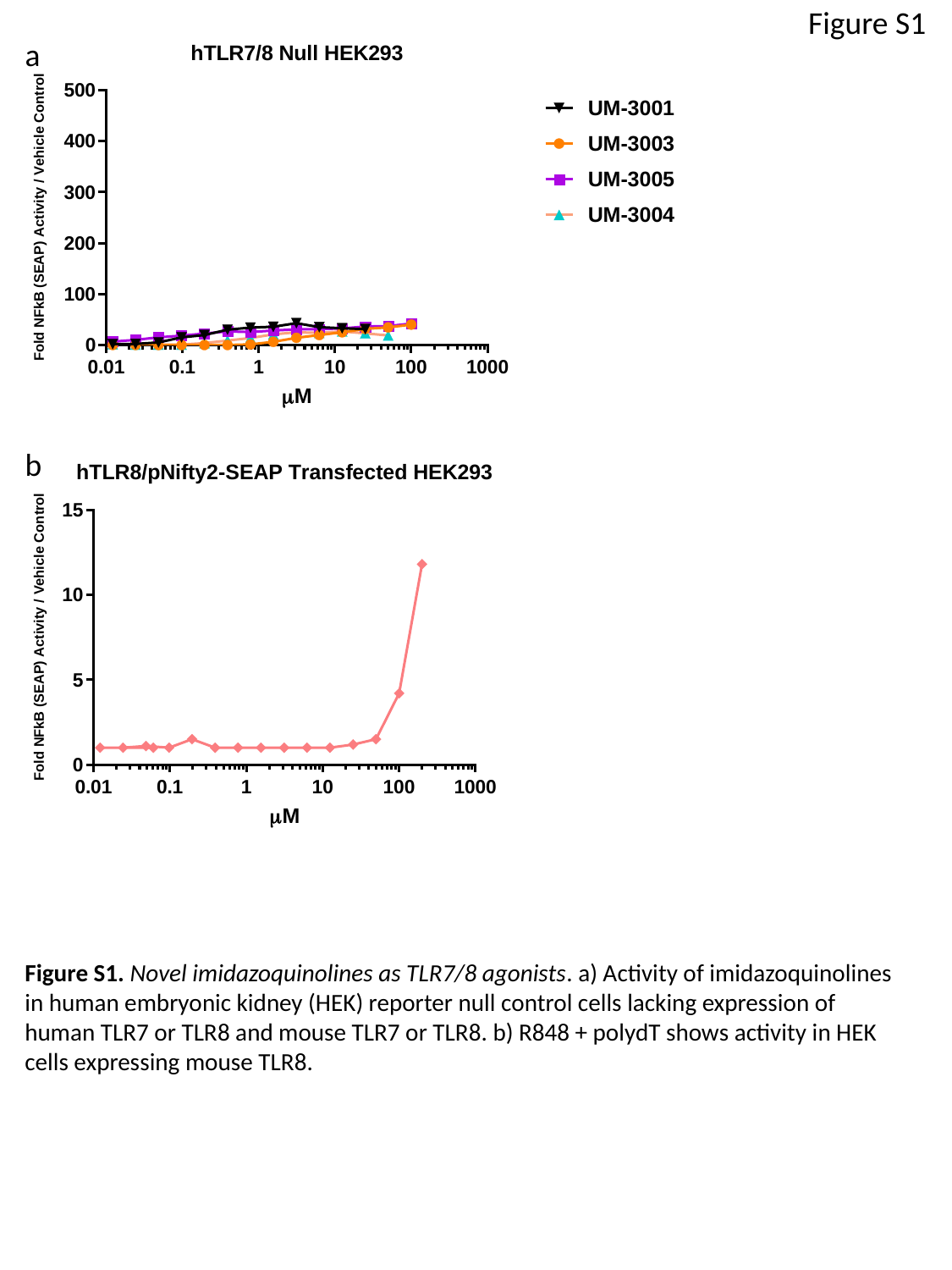

Figure S1
a
b
Figure S1. Novel imidazoquinolines as TLR7/8 agonists. a) Activity of imidazoquinolines in human embryonic kidney (HEK) reporter null control cells lacking expression of human TLR7 or TLR8 and mouse TLR7 or TLR8. b) R848 + polydT shows activity in HEK cells expressing mouse TLR8.

## Slide 3
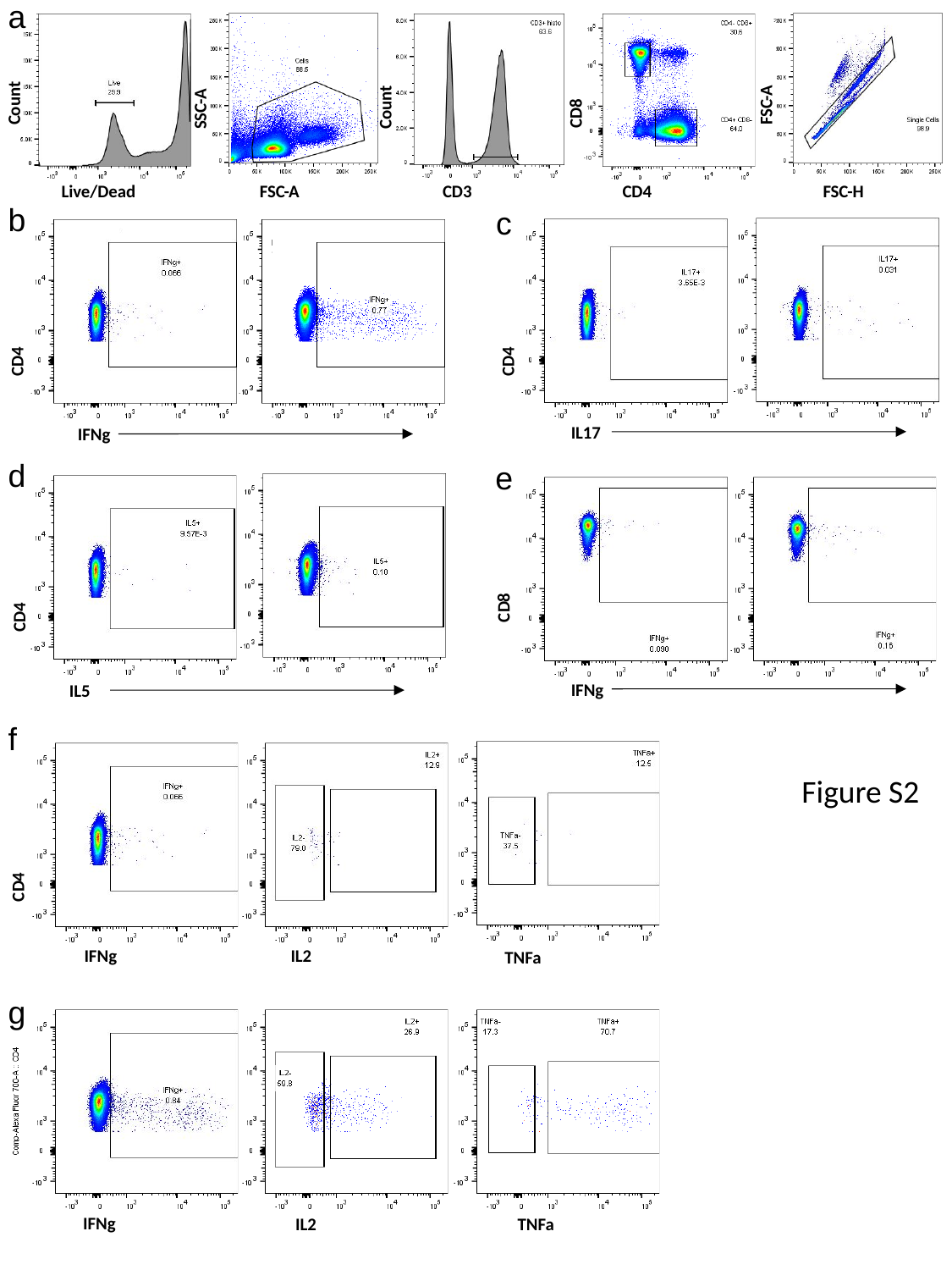

a
CD8
Count
FSC-A
SSC-A
Count
Live/Dead
FSC-A
CD3
CD4
FSC-H
b
c
CD4
CD4
IL17
IFNg
d
e
CD8
CD4
IFNg
IL5
f
Figure S2
CD4
IFNg
IL2
TNFa
g
IFNg
IL2
TNFa

## Slide 4
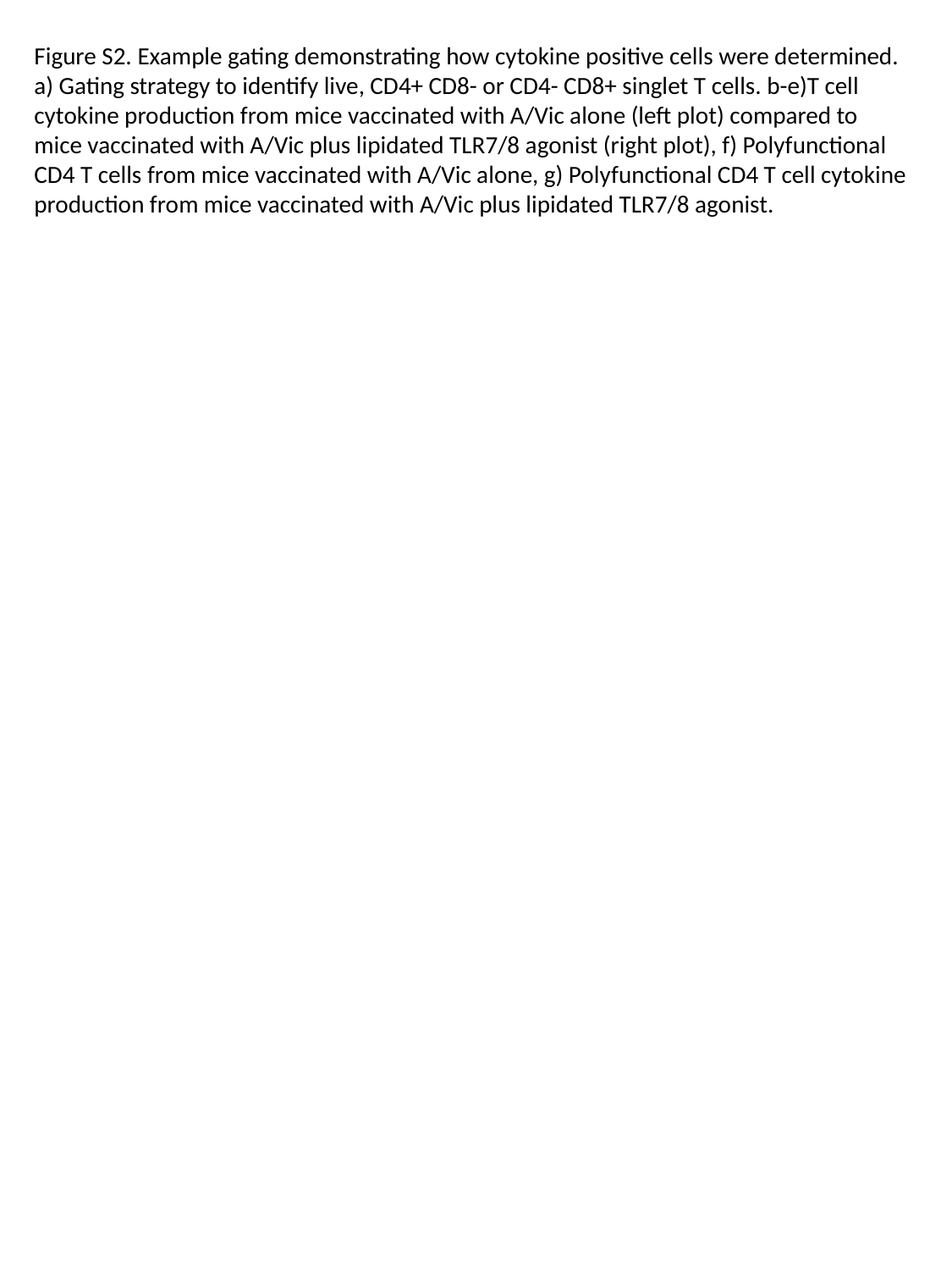

Figure S2. Example gating demonstrating how cytokine positive cells were determined. a) Gating strategy to identify live, CD4+ CD8- or CD4- CD8+ singlet T cells. b-e)T cell cytokine production from mice vaccinated with A/Vic alone (left plot) compared to mice vaccinated with A/Vic plus lipidated TLR7/8 agonist (right plot), f) Polyfunctional CD4 T cells from mice vaccinated with A/Vic alone, g) Polyfunctional CD4 T cell cytokine production from mice vaccinated with A/Vic plus lipidated TLR7/8 agonist.

## Slide 5
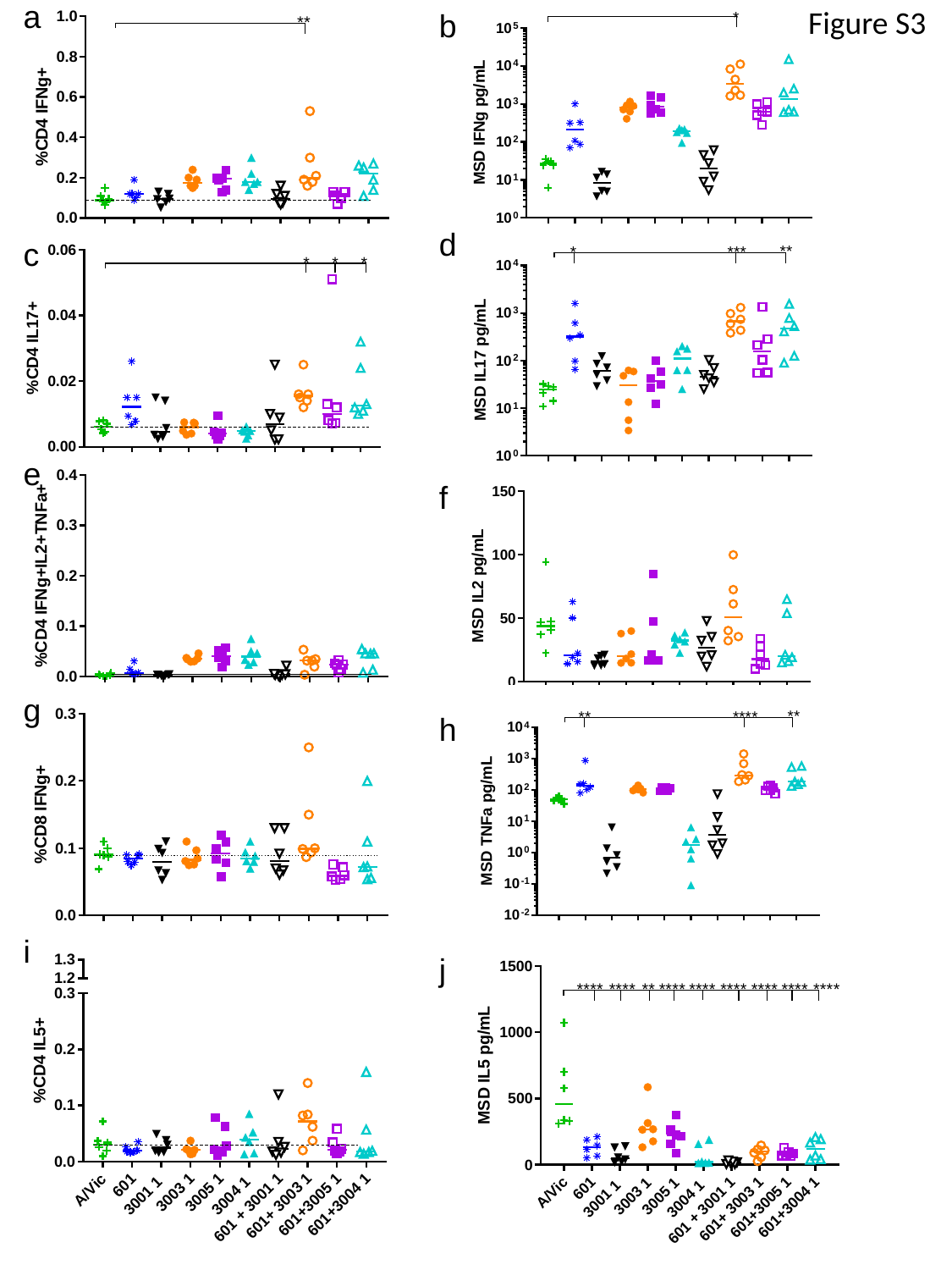

b
a
Figure S3
d
c
e
f
g
h
i
j

## Slide 6
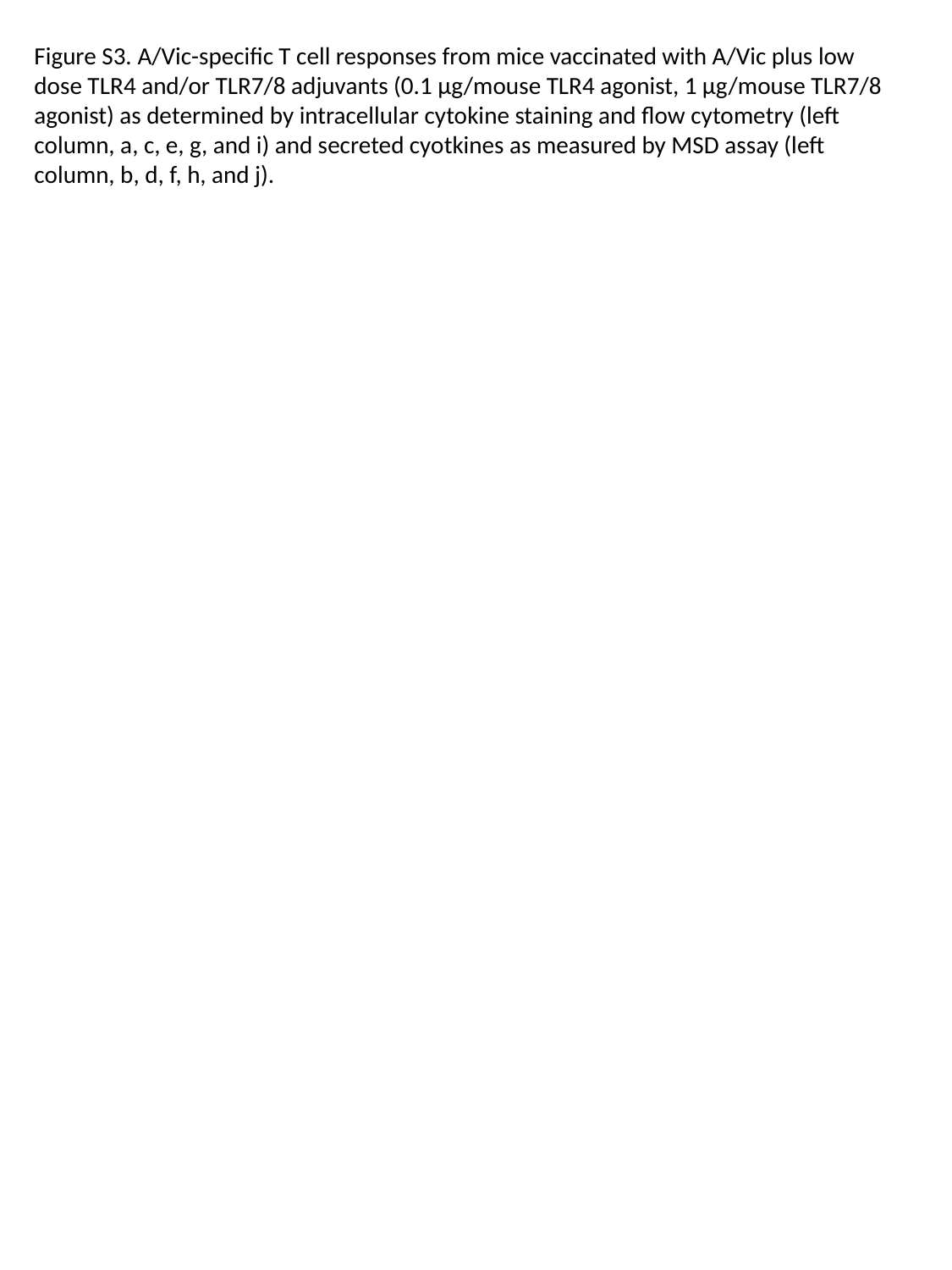

Figure S3. A/Vic-specific T cell responses from mice vaccinated with A/Vic plus low dose TLR4 and/or TLR7/8 adjuvants (0.1 µg/mouse TLR4 agonist, 1 µg/mouse TLR7/8 agonist) as determined by intracellular cytokine staining and flow cytometry (left column, a, c, e, g, and i) and secreted cyotkines as measured by MSD assay (left column, b, d, f, h, and j).

## Slide 7
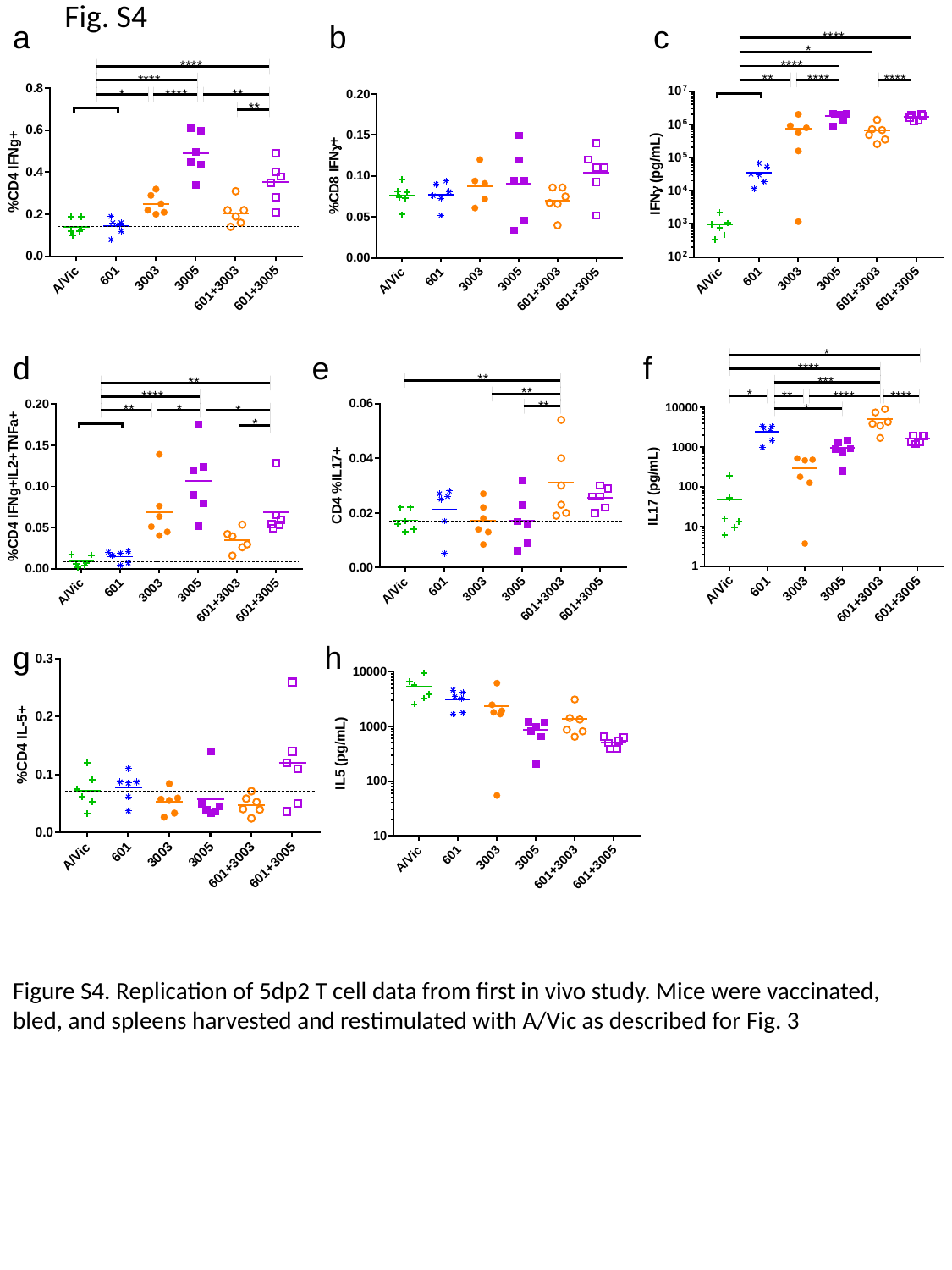

Fig. S4
a
b
c
d
e
f
g
h
Figure S4. Replication of 5dp2 T cell data from first in vivo study. Mice were vaccinated, bled, and spleens harvested and restimulated with A/Vic as described for Fig. 3

## Slide 8
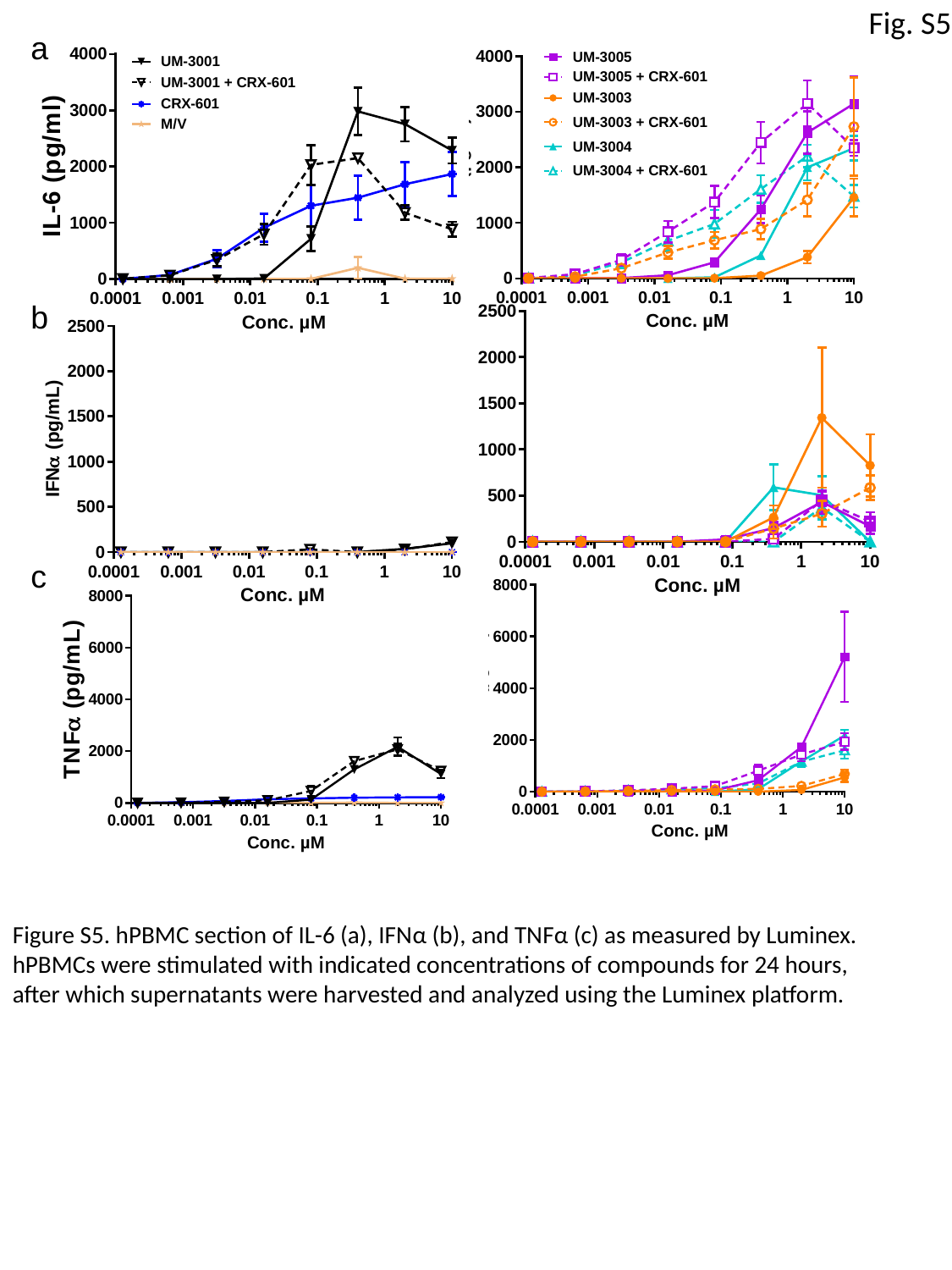

Fig. S5
a
b
c
Figure S5. hPBMC section of IL-6 (a), IFNα (b), and TNFα (c) as measured by Luminex. hPBMCs were stimulated with indicated concentrations of compounds for 24 hours, after which supernatants were harvested and analyzed using the Luminex platform.

## Slide 9
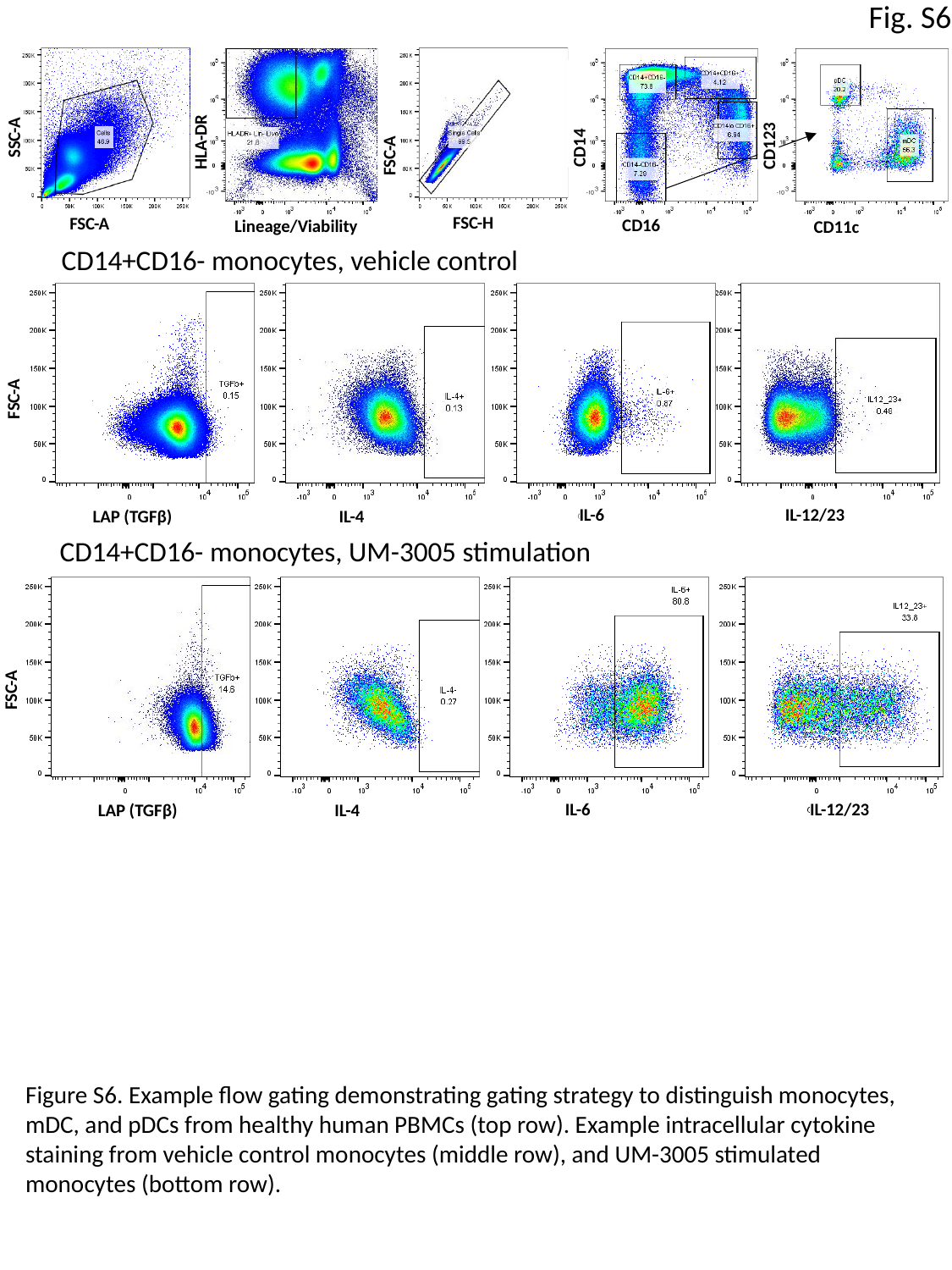

Fig. S6
SSC-A
CD14
CD123
FSC-A
HLA-DR
FSC-H
FSC-A
CD16
Lineage/Viability
CD11c
CD14+CD16- monocytes, vehicle control
FSC-A
IL-6
IL-12/23
LAP (TGFβ)
IL-4
CD14+CD16- monocytes, UM-3005 stimulation
FSC-A
IL-6
IL-12/23
LAP (TGFβ)
IL-4
Figure S6. Example flow gating demonstrating gating strategy to distinguish monocytes, mDC, and pDCs from healthy human PBMCs (top row). Example intracellular cytokine staining from vehicle control monocytes (middle row), and UM-3005 stimulated monocytes (bottom row).
